# Pharmacokinetics of bleomycin in dogs treated with electrochemotherapy

**DOI:** 10.1080/01652176.2026.2648249

**Published:** 2026-03-25

**Authors:** Nina Milevoj, Urša Lampreht Tratar, Tina Kosjek, Mojca Kržan, Filippo Torrigiani, Gregor Serša, Maja Čemazar, Nataša Tozon

**Affiliations:** a Veterinary Faculty, University of Ljubljana, Ljubljana, Slovenia; b Current address: Royal (Dick) School of Veterinary Studies, University of Edinburgh, Roslin, United Kingdom; c Institute of Oncology Ljubljana, Ljubljana, Slovenia; d Jozef Stefan Institute, Ljubljana, Slovenia; e Jozef Stefan International Postgraduate School, Ljubljana, Slovenia; f Faculty of Medicine, University of Ljubljana, Ljubljana, Slovenia; g Istituto di Ricerche Biomediche “Antoine Marxer” - RBM, Turin, Italy; h Faculty of Health Sciences, University of Primorska, Slovenia

**Keywords:** Bleomycin, pharmacokinetics, electrochemotherapy, dogs, electroporation, serum, tumours, veterinary

## Abstract

Bleomycin (BLM) is a cytotoxic antibiotic used in veterinary oncology, primarily in electrochemotherapy (ECT), a local ablative therapy where electric pulses increase drug uptake in tumours. BLM is administered intravenously or intratumourally, with the standard intravenous dose for dogs being 15,000 IU/m², followed by electric pulses 8–10 minutes later. This protocol is derived from human oncology and lacks extensive pharmacological data in dogs. We studied BLM pharmacokinetics in 29 dogs with various tumours treated with intravenous BLM and ECT between 2017 and 2023. Samples were collected from serum of 15 dogs, serum and tumours of 8 dogs, and tumours of 6 dogs. The mean volume of distribution (Vd) of BLM was 224.5 ± 75.02 ml/kg, clearance (CL) was 7.04 ± 2.05 ml/kg/min, and area under the curve (AUC) was 65.87 ± 2.11 µg·min/l. The half-life (t₁_/_₂) of BLM in dogs was 22.03 ± 0.88 minutes. No significant difference was found in tumour BLM concentrations between 8 minutes post-administration and 2 minutes after pulse completion. These results support the recommended 8–28-minute window for applying electric pulses following intravenous BLM administration and may indicate no need for dose adjustments based on body weight or age.

## Introduction

Bleomycin (BLM) is a cytotoxic polypeptide antibiotic produced by *Streptomyces verticullus* (Umezawa et al. 1966). It is a mixture of more than ten components, with bleomycin A_2_ and bleomycin B_2_ as the predominant constituents (Froudarakis et al. 2013). Because of its hydrophilic nature, BLM has severely limited ability to transition into the cell cytoplasm; it is unable to enter the cell by passive diffusion through the cell membrane and enters the cell only via endocytosis (Poddevin et al. 1991; Pron et al. 1999). Within the cell, BLM exerts its cytotoxic activity through oxidation of the deoxyribose of thymidylate and other nucleotides, which causes single- and double-strand breaks in DNA, chromosomal aberrations, gaps, fragments and translocations, ultimately leading to apoptosis (Twentyman 1983). BLM is degraded by bleomycin hydrolase (BLMH), an enzyme found in various tissues. The dose-limiting toxicity of BLM in humans is pulmonary fibrosis and skin toxicity (e.g. desquamation, hyperpigmentation, pruritic erythema). The specific locations of BLM toxicity are due to the relative lack of BLMH in pulmonary and skin tissues (Wittenburg and Gustafson 2018). The presence of BLMH in tumour tissue is also one of the main resistance mechanisms of tumour cells to BLM (Froudarakis et al. 2013). In people, 60–70% of BLM is excreted through the kidneys (Plumb 2018). BLM has occasionally been used as an adjunctive treatment for lymphomas, oral squamous cell carcinomas, teratomas and nonfunctional thyroid tumours in dogs and cats (Plumb 2018). Currently, the use of BLM in veterinary chemotherapy is very limited; a protocol describing its intralesional application for the treatment of acanthomatous ameloblastoma in dogs has been proposed recently with good results (Kelly and Belding, 2010), and a recent paper reported its use in combination with carboplatin for management of dogs with malignant epithelial neoplasms (Giuliano and Almendros 2022).

However, BLM is one of the two most used cytotoxic drugs with electrochemotherapy (ECT), where it can be administered intravenously or intratumourally. In addition to treating tumours with ECT, electosclerotherapy with BLM recently been used in humans for treatment of vascular anomalies (Muir et al. 2023). In ECT, electric pulses are applied locally to the tumours to increase the uptake of the drug due to the increased permeabilisation of the cell membrane (Mir and Orlowski, 1991). When BLM is applied intravenously in dogs treated with ECT, the dose is 15,000 IU/m^2^, which is delivered via slow bolus injection, and electric pulses are applied 8 to 10 minutes thereafter (Tellado et al. 2022). This dose and timeframe are extrapolated from human oncology, where the timeframe of 8–28 minutes has traditionally been used basing on the observed antitumour effect in seven patients (Domenge et al. 1996).

A novel analytical method based on liquid chromatography coupled with tandem mass spectrometry has been used for the detection of BLM in serum and tumour tissue (Kosjek et al. 2016). Using this method, BLM pharmacokinetic parameters were determined in elderly human patients treated with ECT (Grošelj et al. 2016). According to these results, a reduced dose of BLM and a longer therapeutic window (8 to 40 minutes post-BLM injection) are recommended (Grošelj et al. 2016; Gehl et al. 2018). Preliminary results showed that ECT with a reduced dose of BLM has an antitumour effect comparable to that achieved with a standard BLM dose (Grošelj and Bošnjak, 2018; Jamšek et al. 2020).

In veterinary medicine, the only pharmacokinetic study on BLM was performed almost five decades ago on five healthy Beagle dogs (Strong et al. 1979), but since then, there have been no studies using modern detection methods or on a larger cohort of veterinary patients.

Therefore, the aim of this prospective, nonrandomized study was to determine the pharmacokinetic properties of BLM in dogs treated with ECT to define an effective therapeutic window for ECT. Furthermore, we wanted to evaluate the effects of age and body weight on pharmacokinetic parameters to determine whether adjusting the dose of BLM according to the age and body weight of the dogs should be considered.

## Materials and methods

### Animals

We determined the pharmacokinetic parameters of BLM in the serum and tumours of dogs with histologically different tumours that were treated with intravenously administered BLM, followed by ECT. Animals were included in the study when, according to operating procedures for ECT (Tozon et al. 2016), the treatment protocol consisted of intravenous application of BLM and when owners gave written consent for participation in the clinical study. The study was reviewed and approved by the National Ethics Committee at the Administration of the Republic of Slovenia for Food Safety, Veterinary, and Plant Protection (U34401-19/2021/8). For inclusion, dogs had to have a cytological or histopatholopgical diagnosis of neoplasia, life expectancy of at least three months, no previous treatment with immunosuppressive drugs, no significant co-morbidities, and American Society of Anesthesiologists (ASA) Physical Status classification of I or II (Portier and Ida 2018).

To assess the impact of size and age on pharmacokinetic parameters, we divided the dogs included in the analysis of BLM in the serum into groups according to their:

a) Body weight: Dogs with a body weight ≤25 kg were classified into the group of small dogs, and those with a body weight >25 kg were classified into the group of large dogs. The body surface area (BSA) was also calculated for each dog (Price and Frazier 1998), and a 9-score body condition score (BCS) applied to each patient (Laflamme 1997).

b) Size-related age: Dogs were classified into two age groups (i.e. elderly and non-elderly) adjusted according to their body weight. The classification was adapted from Cattai et al. (2019) and is presented in Table 1.

**Table 1.** Classification of dogs according to size-related age.

Group	Body weight (kg)	Age (years)	Number of patients
Elderly dogs	≤9	≥11	5
	9–25	≥10	
	25–40	≥9	6
	>40	≥8	
Non-elderly dogs	≤9	<11	6
	9–25	<10	
	25–40	<9	6
	>40	<8	

### Treatment protocol

The treatment was performed with the patients under general anaesthesia. Prior to the first treatment, a complete blood count with a differential white blood cell count (ADVIA 120, Siemens, Munich, Germany) was performed. Serum biochemical parameters (glucose, blood urea nitrogen (BUN), creatinine, alanine aminotransferase (ALT), aspartate aminotransferase (AST), alkaline phosphatase (AP), electrolytes (sodium, potassium, chloride), creatine kinase, total proteins, albumin, and pancreatic lipase) were determined with an automatic biochemical analyser (RX Daytona, Randox, Crumlin, Great Britain). Diagnostic imaging (thoracic radiographs and abdominal ultrasound) and evaluation of draining lymph nodes for staging purposes were performed at the clinician’s discretion.

The dogs were premedicated with an intravenous administration of midazolam (Midazolam Torrex, Torrex Pharma GesmbH, Vienna, Austria; 0.2 mg/kg), and general anaesthesia was induced via propofol (Diprivan, Zeneca, Grangemouth, United Kingdom; 3–6 mg/kg) and maintained with isoflurane (Isoflurin, Vetpharma Animal Health, Barcelona, Spain) mixed within 100% oxygen. During anaesthesia, dogs received intravenous fluids of Hartmann's solution (B. Braun Melsungen AG, Melsungen, Germany) at a rate of 5 ml/kg/h.

After general anaesthesia has been established, BLM (Bleomycinum, Heinrich Mack. Nachf. GmbH, Illertissen, Germany) was administered in a slow bolus (2 minutes) at a dose of 15,000 IU/m^2^. Eight minutes after BLM administration, electrical pulses were delivered to the tumour (pulse generator: Cliniporator, IGEA S.r.l., Carpi, Italy).

The type of electrode was selected for each patient individually; in the case of small and superficial tumours, plate electrodes (IGEA S.r.l.,) were used, whereas for deeper, larger, or infiltrative tumours, needle row or hexagonal electrodes (IGEA S.r.l.,) were used. The parameters for pulse delivery were as follows: 8 unipolar electric pulses of 100 µs duration, an amplitude-to-electrode distance ratio of 1300 V/cm and a frequency of repetition of 5 kHz. The treatment was performed according to standard operating procedures for ECT in veterinary medicine (Tozon et al. 2016).

### Blood and tumour samples collection

One mililiter of whole blood was collected from the cephalic vein into serum-separating tubes (VACUETTE® TUBE 1 ml CAT Serum Clot Activator, Greiner Bio One International GmbH) at time point 0 (before BLM administration) and at 5, 10, 20, 30 and 60 or 120 minutes after BLM bolus injection. The samples were centrifuged for 10 minutes at 1300 rpm, and the serum was pipetted into a centrifuge tube, frozen and stored at –20 °C until further analysis. The tumour samples of viable tumour tissue (avoiding areas of macroscopic ulceration and necrosis) were collected with a 4-mm punch biopsy instrument 8 minutes after finishing the administration of BLM (prior to delivery of electric pulses) and 2 minutes after the termination of pulse delivery. The samples were weighed with a precision scale (CPA224S, Sartorius, Gottingen, Germany) and immediately stored at –20 °C until further analysis.

### Sample preparation

Serum samples were prepared for analysis via solid-phase extraction using Oasis HLB 30 mg/1 cc cartridges (Waters Corp., Milford, MA, USA). The cartridges were preconditioned with 1 ml of methanol and equilibrated with an equal volume of 0.1% formic acid. Fifty microliter samples were diluted to 3 ml with 0.1% formic acid and filtered through 0.45-μm cellulose acetate filters. This was followed by sorption on a polymeric sorbent, which was then dried under vacuum for 30 minutes and subsequently eluted using 0.5 ml of Milli-Q water/methanol (6/4) and twice with 0.5 ml of acetonitrile solution. The sample extracts were blown down to 1 ml using nitrogen and then kept frozen until LC–MS analysis, which was performed within 1 month. Epirubicin at a final concentration of 50 ng/ml was used as an internal standard and added to the sample extracts immediately prior to analysis.

Tumour samples of known mass were cooled with liquid nitrogen in a mortar and ground to a fine powder. The homogenised material was quantitatively transferred into 10-ml polypropylene centrifuge tubes by rinsing the mortar six times with 1-ml aliquots of 0.1% formic acid. The tubes were protected from light and placed in an ultrasonic bath for 60 minutes to extract BLM into the aqueous phase. The extracts were centrifuged for 20 minutes at 9000 × g, and the supernatants were filtered through 0.45-μm cellulose acetate syringe filters (Sartorius, Göttingen, Germany).

The filtrates were subjected to solid-phase extraction using Oasis HLB 30 mg/1 cc cartridges, previously conditioned with 1 ml methanol and equilibrated with 1 ml of 0.1% formic acid. The eluates were evaporated to approximately 1 ml under a gentle stream of nitrogen and stored at −20 °C until analysis. Immediately prior to instrumental analysis, 25 μL of of 2 μg/ml epirubicin was added.

### Instrumental analysis

Quantitative analysis was performed with an ultra-performance liquid chromatograph (Nexera X2, Shimadzu Corp., Japan) coupled to a hybrid quadrupole orthogonal acceleration time-of-flight mass spectrometer (QTRAP® 4500, Sciex, Germany). The UPLC system was equipped with a binary solvent delivery system and an autosampler. The injection volume was 5 μl. Separation was achieved at 40 °C by using a 5-cm-long Acquity UPLC® BEH Amide (Waters Corp.) column with a 1.7 μm particle size and 2.1 mm internal diameter. The mobile phases were (A) 10 mM ammonium formate with 0.1% formic acid and (B) acetonitrile. The gradient started with 95% B, which was decreased to 50% in 2 minutes, increased back to 95% in 0.5 minutes and maintained until 6 minutes. The flow rate was 0.3 ml/min. The total separation time was 6 minutes, with BLM elimination at 2.5 minutes. BLM was analysed via electrospray ionisation in positive mode. The capillary voltage was set to 5.5 kV. We determined the copper complex of the A_2_ BLM fraction, namely, its quantitative transition (738.4 > 707.2) and two confirmatory transitions (738.4 > 295.0 and 738.4 > 514.7). For the copper complex of fraction B_2_, we monitored the quantitative transition 743.8 > 707.2 and two confirmatory transitions: 743.8 > 551.2, 743.8 > 334.0.

The validation of the analytical method for determining BLM in serum confirmed the inherent selectivity, sensitivity (lower limit of detection of 1 ng/ml), linearity (R^2^ of 0.99), precision (injection repeatability RSD of 0.6%, method repeatability RSD of 7.4%) and accuracy (±6.1%). For tumour tissue, the lower limit of detection was 50 ng/g at a sample mass of 100 mg, with precision (injection repeatability RSD = 2.1%; method repeatability RSD = 7.0%) and accuracy (±8.9%).

### Pharmacokinetic data analysis

We determined the following pharmacokinetic parameters:

The *C*_*0*_ (concentration of BLM in the serum at time point 0) value was extrapolated from the natural logarithm (ln) concentration versus time plot for each pooled data set. Next, the area under the serum concentration‒time curve (AUC) was calculated via the trapezoid method from time 0 to infinity (∞) (AUC_0→__∞_), and plasma clearance (CL) was calculated via the following equation:

$$\mathrm{CL}=\mathrm{dose}(\mathrm{mg})/{\mathrm{AUC}}_{0\to \infty }(\mathrm{mg}\times \;\min\;/l)$$

The elimination rate constant (k_el_) was determined from the plot of the ln concentration versus time curve, and the half-time was calculated as:

$$t_{1/2}=0.693/k_{\mathrm{el}}$$

The volume of distribution (Vd) was calculated via the following equation:

$$\mathrm{Vd}=\;\;\;\;\;t_{1/2}(\min)\times \mathrm{CL}(l/\;\min)/0.693$$

### Tumour response analysis

The tumour volumes were calculated according to the ellipsoid structure:

a×b×c×π/6

Tumour response to treatment was assessed 1 month after treatment by measuring the longest tumour diameter according to the Response Evaluation Criteria for Solid Tumours in Dogs (VCOG) (Nguyen and Thamm, 2015; Tratar et al. 2023).

### Statistical analysis

For statistical analysis, we used SigmaPlot 11.0 (Systat Software, San Jose, California, USA), IBM Statistics Package SPSS, Version 25 for macOS (IBM Corp., Armonk, New York, USA) and GraphPad Prism 7.05 (GraphPad Software, Inc., La Jolla, California, USA). Descriptive statistics such as frequencies, percentages, means, medians and standard deviations were used to describe and summarise the data. The normality of the distribution was determined by the Shapiro‒Wilk test. The differences between groups were evaluated by t tests or one-way analysis of variance (ANOVA), followed by the Holm‒Sidak test for multiple comparisons. In addition, Pearson’s correlation coefficient was used to determine the statistical relationship between pharmacokinetic parameters, and linear regression was used to investigate the effects of dogs’ demographic data on pharmacokinetic parameters. A *p* value < 0.05 was considered significant.

## Results

### Demographic data

Between May 2017 and April 2023, 29 dogs with histologically different tumours were included in the study; the details on the included dogs is presented in Table 2. For the 29 dogs, we determined the pharmacokinetics of BLM in the serum in 15/29 (51.7%), in the serum and tumours in 8/29 (27.6%), and in the tumours only in 6/29 (29.7%) dogs. The age of the dogs ranged from 5–13 years (median 9 years). Most dogs (21/29, 72.4%) were females, and 8/29 (27.6%) were males. Most dogs (20/29, 69%) were diagnosed with mast cell tumours; 14/20 (70%) had cutaneous mast cell tumours, 5/20 (25%) had subcutaneous mast cell tumours, and one (1/20, 5%) had both cutaneous and subcutaneous mast cell tumours. The remaining dogs were diagnosed with soft tissue sarcomas (5/29, 17.2%), hepatoid gland adenoma, oral malignant melanoma, oral fibrosarcoma and hepatoid gland carcinoma (each 1/29, 3.4%). All dogs had normal serum creatinine levels and no other significant abnormalities in serum biochemistry, except patient no. 4, who had mildly elevated serum creatinine (148.8 mmol/l, reference range 44.2–132.6 mmol/l). The weight of the dogs ranged from 5.9 to 47.0 kg (median 25.0 kg).

**Table 2.** Dogs included in the study.

Patient no.	Breed	Gender	Age (years)	Body weight/BSA (kg/m^2^)/BCS (1–9)	Tumour type
1	American Staffordshire Terrier	FN	6	27.3/0.909/7	Subcutaneous mast cell tumour
2	American Staffordshire Terrier	FN	9	25.0/0.864/6	Subcutaneous mast cell tumour
3	Crossbreed	ME	9	38.0/1.142/7	Oral fibrosarcoma
4	Bullterrier	FN	13	20.0/0.744/7	Cutaneous mast cell tumour
5	Boxer	FN	6	27.0/0.909/6	Cutaneous mast cell tumour
6	French Bulldog	FN	9	14.4/0.587/6	Cutaneous and subcutaneous mast cell tumours
7	Jadgterrier	FN	9	11.3/0.500/6	Cutaneous mast cell tumour
8	Crossbreed	FN	6	12.4/0.529/7	Cutaneous mast cell tumour
9	Cocker Spaniel	ME	10	11.0/0.500/6	Oral malignant melanoma
10	Crossbreed	MN	9	24.5/0.840/6	Soft tissue sarcoma
11	Crossbreed	FN	8	35.7/1.081/8	Subcutaneous mast cell tumour
12	Crossbreed	FN	5	12.6/0.529/6	Cutaneous mast cell tumour
13	Labrador Retriever	MN	9	44.9/1.259/7	Cutaneous mast cell tumour
14	Crossbreed	FN	13	34.9/1.060/7	Cutaneous mast cell tumour
15	Weimaraner	FN	5	29.9/0.953/5	Subcutaneous mast cell tumour
16	Basset Hound	FN	7	29.9/0.953/6	Cutaneous mast cell tumour
17	Giant Schnauzer	MN	10	47.0/1.316/7	Hepatoid gland adenoma
18	Beagle	FN	10	17.3/0.668/6	Soft tissue sarcoma
19	Pit Bull Terrier	MN	11	32.6/1.018/6	Cutaneous mast cell tumour
20	Labrador Retriever	FN	8	32.9/1.018/5	Cutaneous mast cell tumour
21	Braque Saint-Germain	FN	10	29.8/0.953/5	Soft tissue sarcoma
22	Parson Russell Terrier	MN	13	10.0/0.469/6	Subcutaneous mast cell tumour
23	Whippet	FN	11	10.8/0.469/4	Soft tissue sarcoma
24	Shih Tzu	FN	9	8.0/0.404/6	Hepatoid gland carcinoma
25	Boxer	MN	6	39.7/1.175/7	Cutaneous mast cell tumour
26	Crossbreed	FN	5	6.4/0.295/6	Soft tissue sarcoma
27	Maltese	FN	12	5.9/0.333/5	Cutaneous mast cell tumour
28	Crossbreed	FN	13	27.0/0.909/7	Cutaneous mast cell tumour
29	French Bulldog	FN	10	9.9/0.469/6	Cutaneous mast cell tumour

BCS - body condition score, BSA - body surface area, FN - neutered female, ME - entire male, MN - neutered male.

Age, body weight, size-related age and sex were assessed for potential effects on the pharmacokinetics of BLM in the serum. The distribution of dogs included in the analysis of the pharmacokinetics of serum BLM, which was based on their age, weight and size, is presented in Table 3.

**Table 3.** Distribution of 23 dogs included in the serum BLM pharmacokinetics analysis, based on age, weight and size.

Variables	Non-elderly male (*n* = 1)	Non-elderly female (*n* = 11)	Elderly male (*n* = 6)	Elderly female (*n* = 5)
Age (years)				
Mean (SD)	8.30 (.000)	6.99 (1.693)	10.05 (1.394)	11.32 (1.451)
Range	/	5.11–9.60	9.11–12.80	10.00–13.20
Weight (kg)				
Mean (SD)	24.50 (.000)	23.49 (9.060)	30.57 (16.366)	22.55 (9.716)
Range	/	11.30–35.70	10.00–47.00	10.80–34.90
Size				
Large > 25 kg	/	6	4	2
Small ≤ 25 kg	1	5	2	3

SD - standard deviation.

### Serum pharmacokinetic parameters of BLM

**Figure 1.**
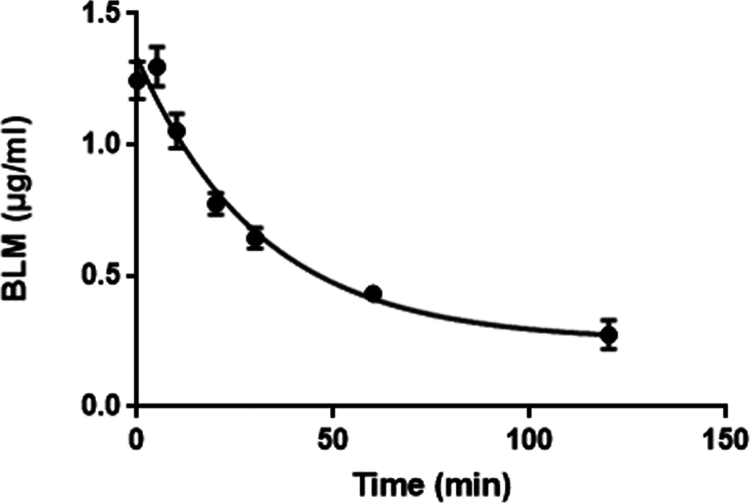
Serum elimination curve of BLM in dogs.

Five minutes after intravenous bolus administration of BLM, the mean serum BLM concentration determined was 1.299 ± 0.075 µg/ml. The serum BLM concentration gradually decreased with elimination rate constant 0.032 min^−1^ and reached 0.433 ± 0.03 µg/ml 60 minutes after administration. Figure 1 shows the monophasic serum elimination curve of BLM in dogs.

The calculated mean Vd was 224.5 ± 75.02 ml/kg, the CL was 7.04 ± 2.05 ml/kg/min or 199.94 ± 57.77 ml/m^2^/min, and the AUC was 65.87 ± 2.11 µg·min/l. The t_1/2_ of BLM in dogs was 22.03 ± 0.88 min.

We found that male dogs had a greater Vd (ml/kg), CL (ml/min/kg), and k_el_ (min^−1^) (mean: 233.96, 7.47, and 0.03348, respectively) than female dogs (mean: 220.36, 6.85, and 0.03219, respectively) and lower t_1/2_ (min) and C_5 min_ (ng/ml) (mean: 21.03 and 1.16) values than female dogs (mean: 22.77 and 1.36). However, the difference was not statistically significant. The results are presented in Table 4.

**Table 4.** Serum BLM pharmacokinetic parameters of patients, based on sex.

Variables	Mean Vd (ml/kg)	Mean CL (ml/min/kg)	Mean t_1/2_ (min)	Mean k_el_ (min^−1^)	Mean C_5min_ (ng/ml)
Males (*n* = 7)	233.96	7.47	21.03	.03348	1.16
Females (*n* = 16)	220.36	6.85	22.77	.03219	1.36

CL - serum clearance, C_5min_ - concentration of BLM in the serum at time point 5 minutes, k_el_ - elimination rate constant, t_1/2_ - elimination half-time, Vd - volume of distribution.

When the dogs were distributed according to their size-related age, the study did not find significant differences between the groups, although the results revealed that the elderly male dogs had higher Vd (ml/kg), CL (ml/min/kg), t_1/2_ (min) and k_el_ (min^−1^) (mean 238.40; 7.57; 21.08; 03348, respectively) and lower C_5min_ (ng/ml) (mean, 1.13) than did the non-elderly male dog. When comparing female dogs, the Vd (ml/kg), CL (ml/min/kg) and k_el_ (min^−1^) values of elderly female dogs were greater (mean 230.45; 7.12; 03415, respectively), and t_1/2_ (min) and C_5min_ (ng/ml) values were lower (mean 21.51; 1.26) than those of non-elderly female dogs (mean 215.78; 6.73; 03130; and 23.34; 1.40, respectively). However, these differences were not statistically significant. The pharmacokinetic parameters of the patients, grouped based on their size-related age, are presented in Table 5.

**Table 5.** Serum BLM pharmacokinetic parameters of patients, based on size-related age.

Variables	Vd (ml/kg)	CL (ml/min/kg)	t_1/2_ (min)	k_el_ (min^−1^)	C_5min_ (ng/ml)
Non-elderly male (*n* = 1)	207.34	6.93	20.73	.03343	1.34
Elderly males (*n* = 6)					
Mean	238.40	7.57	21.08	.03348	1.13
SD	50.93	1.27	3.17	.00533	.16
Median	252.90	7.38	22.13	.03132	1.14
Min	150.29	6.07	17.15	.02860	.84
Max	301.44	9.64	24.23	.04040	1.32
Non-elderly females (*n* = 11)					
Mean	215.78	6.73	23.34	.03130	1.40
SD	78.69	2.68	4.14	.00588	.45
Median	210.67	5.95	23.39	.03000	1.43
Min	114.86	3.01	16.36	.02236	.87
Max	348.37	12.08	31.00	.04236	2.32
Elderly females (*n* = 5)					
Mean	230.45	7.12	21.51	.03425	1.25
SD	107.75	1.57	6.01	.00861	.31
Median	215.96	7.67	19.51	.03553	1.18
Min	126.42	5.15	16.03	.02269	.93
Max	376.12	8.53	30.54	.04325	1.59

CL - serum clearance, C_5min_ - concentration of BLM in the serum at time point 5 minutes, k_el_ - elimination rate constant, SD - standard deviation, t_1/2_ - elimination half-time, Vd - volume of distribution.

Furthermore, Pearson’s correlation was performed to determine the relationships between the age of dogs and the pharmacokinetic parameters. There was a moderate, negative correlation between the age of the dogs and Vd of BLM (ml/kg), which was statistically significant (r = –.455, *n* = 23, *p* = .029), indicating that non-elderly dogs had a greater Vd than did elderly dogs. No statistically significant relationship was found between the weight of the dogs and the pharmacokinetic parameters.

To assess the effects of dogs’ demographic data on pharmacokinetic parameters, linear regression was performed. No significant regression equation was found, except for the age of the dogs and t_1/2_ (F(1, 21) = 5.481, *p* < 0.029), with an R^2^ of 0.207. Our regression output indicates that 20% of variation in t_1/2_ is explained by the increased age of dogs.

### Pharmacokinetic parameters of BLM in tumours

**Figure 2.**
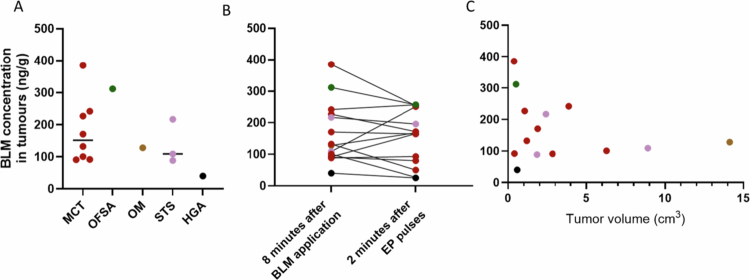
Determination of the BLM concentration in the tumour tissue of different histological types 8 minutes after BLM administration (A), determination of the difference between the first and second sampling time points (B) and the correlation between the tumour volume and the BLM concentration in the tumour 8 minutes after intravenous BLM administration (C); MCT mast cell tumour, OFSA oral fibrosarcoma, OM oral melanoma, STS soft tissue sarcoma, HGA hepatoid gland adenoma.

The BLM concentration measured 8 minutes after intravenous administration ranged from 40 to 385 ng/g, with a median concentration of 130 ng/g. Two minutes after the termination of pulse delivery, the BLM concentration ranged from 27 ng/g to 257 ng/g, with a median concentration of 167 ng/g, regardless of the tumour histological type (Figure 2A).

There was no significant difference in BLM concentration between the two time points of tumour sampling (*p* = 0.7182; Figure 2B). Complete tumour response after the treatment was observed in 6/14 (42.9%) sampled tumours, all of which were mast cell tumours. Additionally, partial response was determined in 5/14 cases (36%), stable disease in 2/14 cases (14%) and progressive disease in 1/14 case (7%). No correlation was detected between the tumour volume and the BLM concentration (Figure 2C) or between the BLM concentration before EP delivery and the response to treatment (*p* = 0.964). Additionally, there was a trend toward a correlation between the serum BLM concentration at the 5-minute time point and that in the tumour tissue 8 minutes after BLM administration (prior to the administration of EP pulses); however, this correlation was not statistically significant (*p* = 0.064). Data on patient details and results of BLM pharmacokinetics in tumours is presented in Table 6.

**Table 6.** Patient data and results of BLM pharmacokinetics in tumours.

Patient no.	Tumour location	Histological type	Tumour volume (cm^3^)	Response 1 month aftertreatment	Bleomycin concentration per 1g of tumour (ng/g) I	Bleomycin concentration per 1g of tumour (ng/g) II
9	Sublingual	Oral malignant melanoma	14.1	PD	128.0	168.0
10	Maxilla	Oral fibrosarcoma	0.5	SD	312.0	257.0
11	Caudal to the ear	Subcutaneous mast cell tumour	0.4	CR	385.0	254.0
14	Right ear	Cutaneous mast cell tumour	1.1	SD	227.0	169.0
16	Left tarsus	Cutaneous mast cell tumour	0.4	CR	91.8	165.0
18	Left elbow	Soft tissue sarcoma	8.9	PR	109.0	251.0
20	Left metatarsus	Cutaneous mast cell tumour	3.9	CR	242.0	257.0
21	Left carpus	Soft tissue sarcoma	2.4	PR	217.0	196.0
24	Perianal	Hepatoid gland carcinoma	0.6	PR	40.2	25.3
25	Left metatarsus	Cutaneous mast cell tumour	2.8	CR	90.9	79.8
26	Right metacarpus	Soft tissue sarcoma	1.8	PR	88.6	93.3
27	Tail	Cutaneous mast cell tumour	6.3	PR	100.7	27.4
28	Right metatarsus	Cutaneous mast cell tumour	1.9	CR	170.6	164.2
29	Right hind leg thigh	Cutaneous mast cell tumour	1.2	CR	132.2	50.4

I - 8 minutes after bleomycin administration, II - 2 minutes after termination of pulse delivery, PD - progressive disease, SD - stable disease, CR - complete response, PR - partial response.

## Discussion

The aim of our study was to determine the pharmacokinetics of BLM in dogs treated with ECT after intravenous bolus administration of BLM. Moreover, we intended to determine whether the time window for pulse delivery (8–28 minutes after BLM administration) is appropriate and whether it is necessary to adjust either the dose of BLM or the time window depending on the age and body weight of the dogs.

To our knowledge, the only study on the pharmacokinetics of BLM in dogs was conducted more than 40 years ago (Strong et al. 1979). In this study, the pharmacokinetic properties of BLM were determined in five healthy experimental dogs via radioimmunoassay (RIA), with a detection limit of 2–5 ng/ml BLM in the serum. After intravenous administration, BLM had a biphasic elimination curve, with a t_1/2_ of 1.01 ± 0.19 h and a Vd of 125 ± 72.5 ml/kg. In our study, the elimination curve of BLM was monophasic, t_1/2_ 22.03 ± 0.88 min. The Vd of BLM was 224.5 ± 75.02 ml/kg. There are several possible explanations for the differences between the results, of which the method used to measure BLM is the most likely. Strong et al. used RIA, whereas in our study, we used a more sensitive method, LC‒MS/MS, with a significantly lower limit of detection (1 ng/ml). The advantages of LC‒MS/MS over RIA are higher specificity and sensitivity and a lower limit of detection (Xu et al. 2014). We studied 23 dogs of different ages and body weights, whereas Strong et al. studied the pharmacokinetics of BLM in five healthy dogs of the same breed with similar weights and ages. In addition, electroporation was not used in the study after BLM administration.

We found that BLM has a low Vd in dogs (Smith et al. 2015), which is consistent with the hydrophilic nature of the drug and the difficult passage of BLM across the cell membrane, with a consequent reduction of sequestration in peripheral tissues. Consequently, BLM also has a short t_1/2_ (22 minutes); the calculated t_1/2_ confirms that the time window for delivery of the electric pulses in ECT is performed while the BLM concentration in the serum is appropriate. In dogs, the therapeutic concentration of BLM for ECT has not yet been determined, but in general, drug plasma concentrations fall below therapeutic levels at 4–5 times the t_1/2_ (Ito 2011), which in our case would mean that BLM is eliminated from the body after 88–110 minutes. We believe that a time window of 8–28 minutes after BLM application is appropriate but could potentially be extended, as in older patients in human oncology. In our study, the dogs had a relatively low CL (7.04 ± 2.05 ml/min/kg) (Toutain and Bousquet-Mélou 2004). One possible explanation for the low CL could be the accumulation of BLM in the tumour tissue to which the electrical pulses were delivered as a result of the ‘vascular lock’ phenomenon, in which vasoconstriction caused by the electric pulses leads to retention of the chemotherapy drug in the tumour (Gehl and Skovsgaard, 2002). However, BLM can also accumulate in the lungs and skin, where it is not metabolised by BLMH, which is why it can cause side effects in these organs in humans (Froudarakis et al. 2013). No such side effects were observed in the treated dogs. However, advanced diagnostic imaging (CT scan) with biopsy and histopathological analysis of the lungs and skin are needed to rule out the accumulation of BLM in these tissues. Nevertheless, the latter would be unacceptable from an ethical point of view.

The most important variables affecting the pharmacokinetics of the drug are age, body weight, organ dysfunction (especially of the kidneys and liver), concomitant use of other drugs and the genetic characteristics of the animal (Joerger 2012). Aging may affect the pharmacokinetics and pharmacodynamics of drugs, but age-related changes in specimens do not occur at the same rate. This is particularly true for dogs, where we see marked differences in size and an inversely proportional relationship between body weight and life expectancy (Galis and Van Der Sluijs, 2007). In veterinary medicine, there is currently no standard that defines age groups for different life stages of dogs. Therefore, we find different classification systems in the literature (Reid 1996; Hayek and Davenport 1998; Lund et al. 1999), but they have the common disadvantage that the age groups do not take into account the body weight or size of the dogs. Therefore, in our study, we adopted the classification of Cattai et al. (2019), where dogs were divided into two age groups (elderly vs. non-elderly dogs) according to their body weight to investigate the pharmacokinetic parameters of propofol.

In elderly dogs, like humans, the loss of liver and kidney function is expected, and considerable changes in body composition occur. As an organism ages, the proportion of total water and lean body mass gradually decreases, leading to a relative increase in body fat percentage. This might influence the distribution parameters of water-soluble drugs such as BLM (Mangoni and Jackson 2004). Reduced Vd and consequently increased serum concentrations of drugs can occur in elderly people. In our study, we were able to demonstrate a moderate negative correlation between the age of the dog and Vd, as younger dogs had higher Vd than older dogs, but we found no differences in CL. These results may be because none of the dogs had significantly impaired renal function, which could delay elimination of BLM from the body. Vd may therefore be increased in older individuals due to a relative increase in body fat percentage, but the drug is excreted from the body at a comparable time to that of younger dogs because of the maintenance of renal function. A tendency for increased t_1/2_ with a relatively low coefficient of determination (R^2^ = 0.207) was also found in elderly dogs. A larger cohort of dogs should be included in the study to confirm or rule out a relationship between the age of the dogs and the higher t_1/2_. In addition, a more sensitive method of measuring renal function (e.g. urinalysis, glomerular filtration rate) should be considered, especially in elderly dogs.

In the literature, we find various classification systems for categorising dogs into size groups according to their body weight. One of the commonly used cutoff points for categorising dogs into large or giant breeds and medium, small or toy breeds is 25 kg (Hawthorne et al. 2004; Su et al. 2015). Differences in pharmacokinetic parameters are to be expected in smaller dogs owing to their faster metabolism. However, no differences were observed between large (>25 kg) and small (≤25 kg) dogs. Following the example of human medicine, some authors suggest that a calculation adapted to the BSA of dogs is more appropriate for pharmacokinetic parameters, especially CL (Goy-Thollot and Chafotte, 2006), as body surface area better reflects the basal metabolic rate and correlates more strongly with kidney size and body water. However, when we compared CL in dogs with different BSA values, we found no differences between large and small dogs. The reason for our observations could be the low variability in the body weights of the dogs. To confirm our observations, it will be necessary in the future to expand the sample to include miniature (<5 kg) and giant (>50 kg) breeds.

In humans, BLM is mainly excreted via the kidneys, so we expected a lower CL and a longer t_1/2_ in dogs with impaired renal function. In our study group, only one dog had mildly elevated serum creatinine, and the remaining dogs had normal kidney values. In this patient, the CL was actually lower than average, which could be due to impaired kidney function attested by elevated creatinine or due to the age of the dog (13 years). However, Crooke et al. (1977) observed a t_1/2_ prolongation in their study only in patients with significantly reduced creatinine clearance. Serum creatinine is an unreliable indicator of the glomerular filtration rate in elderly people. Despite the decrease in the glomerular filtration rate, we often do not find an increase in serum creatinine because aging reduces the production of creatinine due to a decrease in skeletal muscle mass. Therefore, in the elderly population, more reliable indicators of the glomerular filtration rate should be used to monitor renal function more accurately (Fliser 2008). To exclude the influence of renal function on the pharmacokinetic parameters of BLM, a larger sample of dogs and a more sensitive method of measuring renal function are needed.

In addition to assessing pharmacokinetics in serum, we measured BLM concentrations in tumour tissue at two time points: 8 minutes after intravenous administration of BLM and 2 minutes after electric pulse delivery. Our results showed no statistically significant differences in BLM concentrations between histological tumour types, consistent with findings in human patients, where no differences were observed between squamous cell carcinomas and basal cell carcinomas (Grošelj et al. 2021). Furthermore, our study population included not only epithelial tumours but also other histological types (round cell and mesenchymal), which differ significantly in vessel density, among other features. Despite the small number of cases in each group, it is noteworthy that no difference in BLM concentrations was found between these groups. The median BLM concentrations in canine tumours were similar to those in human samples (130 ng/g in canine tumours and 174 ng/g in human samples). In addition, no significant differences were observed between BLM concentrations in tumour samples collected 8 minutes after BLM administration and those collected 2 minutes after electric pulse delivery, which was performed according to the standard operating procedures for ECT between 8 and 28 minutes after BLM administration. All the samples were thus obtained within a time window of 8–28 minutes, confirming that the BLM concentrations were stable and sufficient within this time frame. Furthermore, no correlation was found between tumour volume and BLM concentration, which is consistent with observations in human studies (Grošelj et al. 2021). We also investigated the correlation between the BLM concentration in the serum and that in the tumour. Although there was a tendency for a correlation between the BLM concentration in the serum at the 5-minute time point and that in the tumour tissue 8 minutes after BLM administration, this correlation was not significant. Further studies in a larger cohort of patients are needed to evaluate this correlation.

There are several important limitations to the present study, the main one being the small patient cohort, particularly in some of the age- and body weight-related treatment groups. The limited sample size restricted meaningful statistical analyses assessing the influence of patient body weight and age on BLM pharmacokinetics. To assess potential variables affecting BLM pharmacokinetics, dogs were divided into various age- and weight-related groups, some of which contained only a small number of dogs. Applying a cutoff based on body weight in dogs is challenging due to the existence of various breeds with significant variability in body constitution. In addition, although the body condition score was applied to each patient, the muscle condition score was not uniformly recorded. Older animals, and particularly cancer-bearing dogs, often have reduced muscle mass, which may lead to underestimation of serum creatinine and potentially introduce bias in the pharmacokinetic parameters. However, none of the included patients had severely reduced muscle mass on physical examination alone; therefore, we consider the risk of this type of bias in the present population to be minimal. Nonetheless, the small and uneven group sizes in our population limited the statistical power of the analyses, precluding definitive conclusions. These findings underscore the need for future studies involving larger and more balanced cohorts.

Secondly, tumour sampling was limited to two time points: 8 minutes after BLM administration—a time point widely accepted in both clinical practice and guidelines for starting electric pulse delivery—and 2 minutes after completion of electric pulse delivery (performed within the 8–28-minute window). The latter was included to assess whether intratumoral BLM concentrations remained adequate throughout the recommended treatment interval. The first sample was collected at the exact same time point in all patients, whereas the timing of the second sample varied depending on the number of electric pulses required to achieve complete tumour coverage. Importantly, none of the procedures exceeded 28 minutes for delivery of electric pulses. The primary objective of this study was to evaluate whether the 8–28-minute time frame recommended in veterinary ECT guidelines is appropriate with respect to intratumoral BLM concentration, which our data confirmed.

In conclusion, the results of our study confirm that the time window for delivery of electrical pulses (8–28 minutes) after intravenous BLM administration is appropriate. BLM has a short half-life in dogs, which reduces the possibility of side effects due to the accumulation of BLM in the body. Our results indicate that the dose adjustment of BLM depending on the age and body weight of the dogs may not be needed. However, further studies in different age and weight groups of dogs are warranted.

## Language editing

The language of this manuscript was improved with the assistance of generative AI tools, including Instatex and Rubriq, which were used solely for grammar, spelling, and style refinement. The authors reviewed and approved all edits and take full responsibility for the final text.
